# Posterior Pulmonary Vein Isolation Lesion Spacing and Left Atrial Posterior Wall Narrowing During Pulsed Field Ablation

**DOI:** 10.1002/joa3.70509

**Published:** 2026-10-28

**Authors:** Yuji Saito, Koichi Nagashima, Ryuta Watanabe, Naoto Otsuka, Shu Hirata, Moyuru Hirata, Masanaru Sawada, Hikaru Masuda, Akinori Matsushima, Masato Sakaki, Yasuo Okumura

**Affiliations:** ^1^ Division of Cardiology, Department of Medicine Nihon University School of Medicine Tokyo Japan

**Keywords:** atrial fibrillation, left atrial posterior wall, pulmonary vein isolation, pulsed field ablation, tissue proximity indication

## Abstract

**Background:**

Left atrial posterior wall (LAPW) narrowing after excessive extension of bilateral pulmonary vein isolation (PVI) lines during pulsed field ablation (PFA) may create a proarrhythmic substrate. The CARTO 3‐integrated variable‐loop circular catheter (VLCC) provides real‐time tissue proximity indication (TPI) and ablation‐tag location, but their relationship with LAPW narrowing remains unclear. Therefore, we evaluated associations of TPI status and inter‐tag distance (ITD) with acute LAPW narrowing and whether an ITD‐guided workflow was associated with LAPW preservation.

**Methods:**

Fifty‐two retrospective and 27 prospective atrial fibrillation patients underwent PFA using a VLCC. In the retrospective phase, we evaluated the association between ITD, measured using TPI‐positive tags and all tags, and LAPW narrowing (< 10 mm) and derived a cutoff. In the prospective phase, the ITD‐guided workflow was applied, and we assessed residual LAPW band width.

**Results:**

In the retrospective phase, both TPI‐positive‐tag ITD and all‐tag ITD correlated with LAPW band width (*r* = 0.63 and 0.70, respectively; both *p* < 0.001). All‐tag ITD showed a numerically higher AUC for LAPW narrowing than TPI‐positive‐tag ITD (0.869 vs. 0.795; *p* = 0.099), with an optimal all‐tag ITD cutoff of 16.5 mm. In the prospective phase incorporating the all‐tag ITD cutoff, residual LAPW band widths were wider at the roof (19.4 ± 7.9 vs. 25.1 ± 7.1 mm; *p* = 0.002) and carina (13.6 ± 7.1 vs. 20.8 ± 8.0 mm; *p* < 0.001) than in the retrospective phase.

**Conclusions:**

An all‐tag ITD‐guided workflow was associated with greater preservation of the residual LAPW band width.

## Graphical abstract

In 52 retrospective VLCC‐PFA cases, all‐tag inter‐tag distance (ITD) correlated with residual left atrial posterior wall (LAPW) band width and identified a 16.5‐mm cutoff for predicting LAPW narrowing (< 10 mm). In 27 prospective cases, the all‐tag ITD target of ≥ 16.5 mm was achieved in 90% of LAPW segments and was associated with wider residual LAPW bands at the roof and carina than in the retrospective cohort. All‐tag ITD may provide a practical intraprocedural target for LAPW preservation.

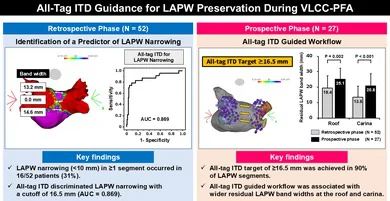

## 1 Introduction

Pulmonary vein (PV) isolation (PVI) is widely accepted as a treatment strategy for atrial fibrillation (AF) [1]. PVI using pulsed field ablation (PFA) is increasingly adopted for AF, offering efficacy comparable to thermal ablation with a favorable safety profile, particularly with respect to esophageal and phrenic nerve injury [2, 3, 4, 5, 6, 7]. Despite these advantages, long‐term clinical success with PFA still depends largely on the quality and spatial distribution of ablation lesions rather than on energy modality alone. In particular, excessive extension of the bilateral PVI lines toward the left atrial (LA) posterior wall (LAPW) may lead to functional narrowing of the LAPW and the creation of slow‐conduction channels that promote LAPW‐origin atrial tachycardia (AT) [8].

In recent years, multiple PFA systems have become available in clinical practice, and device‐specific differences in post‐ablation lesion distribution have been reported [9]. Among these PFA systems, the VARIPULSE Platform (Biosense Webster Inc.), which uses a variable‐loop circular catheter (VLCC) for PVI, has not been adequately evaluated regarding the width of the residual healthy band on the LAPW after PVI. In addition, we have previously reported that catheter‐tissue contact assessed by tissue proximity indication (TPI) software is associated with both acute PVI failure and the durability of ablation lesions [10, 11]. However, the impact of real‐time procedural information, including TPI status and inter‐tag distance (ITD) on preservation of the residual LAPW band width remains unclear.

Therefore, this study investigated the relationship between TPI‐related lesion distribution and LAPW narrowing. We then evaluated whether incorporating the findings obtained from the retrospective analysis into the prospective workflow was associated with preservation of the residual LAPW band width. In addition, acute lesion characteristics according to TPI status were explored as a secondary mechanistic analysis.

## 2 Methods

### 2.1 Study Design

This study was conducted in two phases: a retrospective phase and a prospective phase. In the retrospective phase, we analyzed 58 consecutive AF patients who underwent PFA using a VLCC between April 2024 and April 2025. Six patients were excluded (pre‐existing low‐voltage areas in the LAPW, repeat ablation procedure, planned LAPW isolation, or incomplete 3D mapping data), resulting in a final cohort of 52 patients for analysis. In the prospective phase, we conducted an analysis of 31 consecutive AF patients who underwent PFA using a VLCC between August 2025 and December 2025. Four patients were excluded because of pre‐existing low‐voltage areas in the LAPW, repeat ablation procedures, or planned LAPW isolation. Therefore, 27 patients were included in the prospective phase analysis. This study was conducted according to the Declaration of Helsinki and approved by the Institutional Review Board of Nihon University Itabashi Hospital (RK‐240312‐1).

### 2.2 Retrospective Phase

In the retrospective phase, the electrophysiologic study and anticoagulation followed previously described protocols [9, 11, 12]. For deep sedation, fentanyl and midazolam were administered before femoral venous puncture. Sedation was then maintained with either propofol or continuous dexmedetomidine infusion, with additional fentanyl, propofol, or midazolam administered as needed during ablation to suppress patient movement. Following transseptal puncture, left atrial pre‐mapping was performed using the OCTARAY Mapping Catheter (Biosense Webster Inc.) via a VIZIGO steerable sheath (Biosense Webster Inc.). PFA was performed with a Multi‐Channel PFA Generator (TRUPULSE; Biosense Webster Inc.), a CARTO 3 system (version 8.0, Phase 2.1), and an irrigated VLCC (irrigation rate; 4 mL/min), as described previously [2, 9, 11]. The standard clinical workflow recommends delivering at least 12 applications per PV targeting the PV ostium and antrum [2, 3, 11]. Based on our preclinical and clinical findings, [10, 11] TPI‐positive contact was maximized, and additional applications were delivered at TPI‐negative sites to create transmural lesions. In this study, first‐pass PVI was defined as the completion of the standard 48 applications plus TPI‐guided additional applications delivered before post‐mapping. Post‐ablation high‐density mapping was performed using the OCTARAY Mapping Catheter. Touch‐up PFA was performed for PV gaps identified by high‐density post‐mapping to achieve complete PVI. Other arrhythmias were treated as needed.

### 2.3 Measurement Parameters in the Retrospective Phase

PVI lesions were defined as areas with bipolar voltage < 0.1 mV. The LAPW was divided into three segments (roof, carina, and bottom), and the residual LAPW band width (bipolar voltage ≥ 0.5 mV between bilateral PVI lines) was quantified (Figure 1). Based on a previous report linking a residual healthy band width < 10 mm with slow conduction, [13] such regions were labeled “LAPW narrowing”.

**FIGURE 1:**
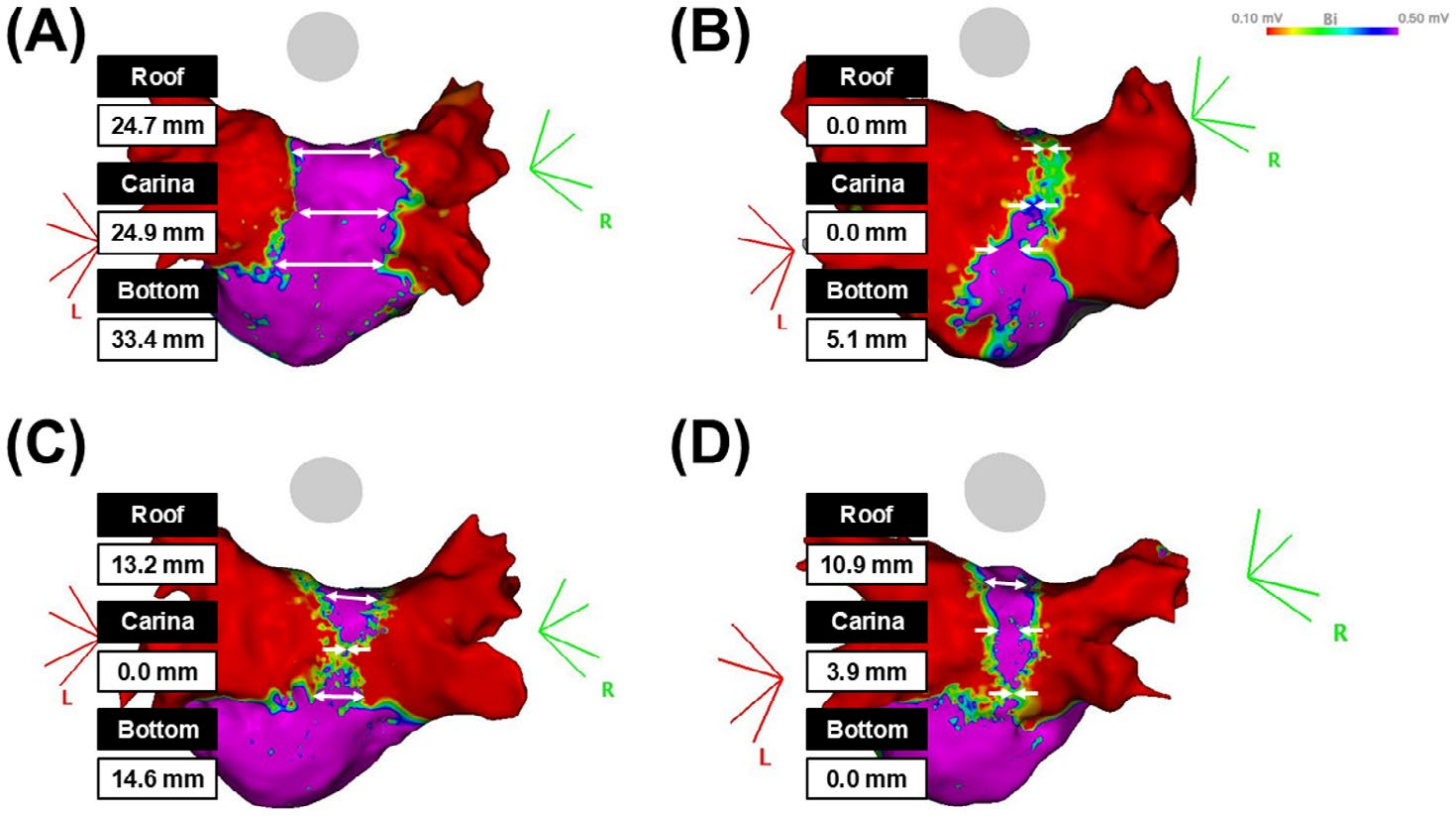
Representative LAPW voltage maps after PVI. Bright purple indicates high voltage (≥ 0.5 mV), and red indicates low voltage (< 0.1 mV). (A) Wide bands in all segments. (B) LAPW narrowing at the roof, carina, and bottom. (C) LAPW narrowing at the carina. (D) LAPW narrowing at the carina and bottom. LAPW; left atrial posterior wall, PVI; pulmonary vein isolation.

In addition, LAPW anatomy was assessed on the pre‐ablation 3D mapping geometry. Roof and bottom lines were drawn between the most superior and inferior points of the bilateral PV‐LA junctions, respectively, with endpoints at the points of maximal inflection between the PV and LA walls [9, 14]. In patients with a left common PV, the superior and inferior margins of the common left PV were used as the left‐sided endpoints. Their average width was defined as the mean anatomical LAPW width, and the area enclosed by these lines and their lateral connections as the LAPW surface area (Figure S1A).

ITD between the bilateral PVI lines was used as a spatial measure of TPI‐related lesion distribution on the LAPW and was calculated for TPI‐positive tags and all tags (regardless of TPI status). The optimal cutoff value of ITD for predicting narrowing (< 10 mm) was calculated to guide the prospective workflow.

### 2.4 Prospective Phase

The prospective workflow incorporated ITD control, with the retrospectively derived cutoff used as a practical target during PVI procedure. The CARTO 3 system (version 8.1, Phase 3.0) was used for real‐time lesion visualization and guidance of lesion placement. In addition, the irrigation rate was increased to 30 mL/min [15, 16]. Pre‐ablation LA geometry was created using either a SOUNDSTAR Ultrasound Catheter (Biosense Webster Inc.) with the SOUND FAM function or the OCTARAY Mapping Catheter. After completion of PFA, post‐ablation high‐density mapping was performed using the OCTARAY Mapping Catheter. Anatomical LAPW measurements were obtained from the post‐ablation high‐density mapping geometry using the same definitions as in the retrospective phase. Touch‐up PFA was performed for PV gaps identified on post‐ablation high‐density mapping to achieve complete PVI. Residual LAPW band width was assessed at the roof, carina, and bottom segments using the same definitions as in the retrospective phase. ITD was measured at each segment, and achievement of the retrospectively derived cutoff was assessed. The relationship between achieved ITD and residual LAPW band width was also evaluated.

### 2.5 Follow‐Up

Patients in both the retrospective and prospective phases were followed after the ablation procedure. Follow‐up visits with 12‐lead ECGs were scheduled at 1, 3, 6, and 12 months, with 24‐h or 2‐week Holter monitoring at 3–6 and 12 months. Recurrence was defined as any atrial tachyarrhythmia lasting > 30 s, excluding events within the 8‐week blanking period [1].

### 2.6 Assessment of Repeat Procedures

Four patients underwent repeat ablation in the retrospective phase. Residual LAPW band widths at the corresponding sites were reassessed using pre‐ablation mapping during the repeat procedure. Changes in residual LAPW band width from the index to the repeat procedure were evaluated, including according to the TPI status of the boundary tags. Recurrence mechanisms were assessed using 3D mapping during the repeat procedure.

### 2.7 Exploratory Mechanistic Analysis

In the prospective phase, an exploratory mechanistic analysis was performed in 5 patients whose LA geometry was created using the SOUND FAM function. A baseline bipolar voltage map was created using the VLCC before PVI [17]. Each PV then underwent a single ablation set (3 applications) with the VLCC, followed by repeat bipolar voltage mapping using the same VLCC without catheter exchange. We evaluated the voltage reduction (pre‐ vs. post‐ablation) in both groups and compared the post‐ablation voltage between TPI‐positive and TPI‐negative tags. Among TPI‐positive tags, post‐ablation voltage was also evaluated according to the number of TPI‐positive energy deliveries (1/3, 2/3, or 3/3). After completion of the mapping, PFA was continued to achieve complete PVI.

### 2.8 Statistical Analysis

Continuous variables were summarized as mean ± standard deviation or as median with interquartile range (25th‐75th percentiles) and compared using Student's *t* test or the Mann–Whitney *U* test, as appropriate. Categorical variables were reported as counts (percentages) and compared with *χ* ^2^ test; when any expected cell count was < 5, Fisher's exact test was applied. Residual LAPW band width among the roof, carina, and bottom segments was compared using a linear mixed‐effects model accounting for within‐patient correlations, followed by Tukey‐adjusted pairwise comparisons. We evaluated the correlation between the ITD of (i) TPI‐positive tags or (ii) all tags and residual LAPW band width. To confirm the robustness of these associations, additional linear mixed‐effects models accounting for segment and within‐patient correlations were performed. In addition, receiver operating characteristic (ROC) analysis was performed to identify predictors of LAPW narrowing (< 10 mm), with comparison of areas under the curve (AUC) where appropriate. Paired Student's *t* tests were used for within‐site changes in bipolar voltage and residual LAPW band width between index and repeat procedures. Separate analyses of covariance were performed to compare post‐ablation bipolar voltage adjusted for pre‐ablation voltage according to TPI status (negative vs. positive) and, among TPI‐positive tags, the number of TPI‐positive energy deliveries (1/3, 2/3, or 3/3). Event‐free survival was estimated using the Kaplan–Meier method. We performed all analyses using JMP software, and *p* < 0.05 was considered significant.

## 3 Results

### 3.1 Baseline Characteristics

Baseline characteristics of the retrospective (*n* = 52) and prospective (*n* = 27) cohorts are shown in Table 1. Complete PVI was achieved in all patients in both cohorts, with touch‐up ablation performed as needed. First‐pass PV isolation success did not differ significantly between the two cohorts (*p* = 0.261). Antiarrhythmic drug use was less frequent in the prospective cohort (*p* = 0.011). The numbers of ablations and applications did not differ significantly between cohorts. By contrast, fluoroscopic time and dose were significantly lower in the retrospective cohort because operators intentionally adopted a zero‐fluoroscopy workflow using OCTARAY for geometry creation [12]. In the prospective cohort, limited fluoroscopy was used during SOUNDSTAR‐based anatomy acquisition and catheter manipulation.

**TABLE 1:** Baseline and procedural characteristics.

	Retrospective phase (*n* = 52)	Prospective phase (*n* = 27)	*p*
Clinical characteristics			
Age (years)	64 ± 11	65 ± 12	0.728
Male sex	38 (73%)	22 (81%)	0.407
Body mass index (kg/m^2^)	24 ± 5	23 ± 2	0.728
Paroxysmal AF	45 (87%)	20 (74%)	0.217
AF duration (months)	5 (3.3, 14)	5 (3, 14)	0.716
Hypertension	25 (48%)	13 (48%)	0.995
Dyslipidemia	15 (29%)	3 (11%)	0.075
Diabetes mellitus	10 (19%)	1 (4%)	0.087
Heart failure	11 (21%)	1 (4%)	0.050
History of stroke	4 (8%)	2 (7%)	1.000
Vascular disease	1 (2%)	1 (4%)	1.000
CHADS_2_ score	1 (0, 2)	1 (0, 2)	0.268
CHA_2_DS_2_‐VASc score	2 (1, 3)	2 (0, 2)	0.568
Antiarrhythmic drug use	27 (52%)	6 (22%)	0.011
Class I	16 (31%)	4 (15%)	0.122
Class III	2 (4%)	0 (0%)	0.544
Class IV	11 (21%)	3 (11%)	0.359
Echocardiographic measurements			
Left ventricular ejection fraction (%)	67 ± 9	67 ± 4	0.878
Left atrial diameter (mm)	38 ± 6	38 ± 7	0.827
Left atrial volume (mL)	43 ± 19	41 ± 20^a^	0.678
3D Mapping‐Based LAPW Assessment			
LAPW surface area (cm^2^)	18.1 ± 4.6	16.6 ± 3.1	0.111
Mean anatomical LAPW width (mm)	40.2 ± 7.1	37.9 ± 4.1	0.123
First‐pass PV isolation success	48 (92%)	22 (81%)	0.261
Number of ablations before post‐mapping	23 ± 5	24 ± 3	0.349
Number of applications before post‐mapping	66 ± 13	71 ± 7	0.111
Number of total ablations	24 ± 6	26 ± 4	0.247
Number of total applications	69 ± 16	75 ± 10	0.083
Total fluoroscopic time (min)	0.0 (0.0, 0.2)	1.3 (0.1, 3.8)	< 0.001
Total fluoroscopic dose (mGy)	0.0 (0.0, 0.7)	3.3 (0.0, 17.9)	< 0.001
Primary PV isolation time (min)	31 ± 10	33 ± 11	0.361
Total procedure time (min)	107 ± 29	99 ± 40	0.269
Irrigation rate (mL/min)	4	30	—
3D geometry creation before ablation	OCTARAY	SOUNDSTAR or OCTARAY	—
CARTO 3 version	Version 8.0, Phase 2.1	Version 8.1, Phase 3.0	—

*Note:* Values are shown as the mean ± standard deviation, median (25th‐75th interquartile range) or *n* (%).

Abbreviations: AF, atrial fibrillation; LAPW, left atrial posterior wall; PV, pulmonary vein.

^a^ Analysis was performed on 26 patients due to one missing data point.

In the retrospective cohort, patients were classified by residual LAPW band width: ≥ 10 mm in all segments (“wide group”, *n* = 36) or < 10 mm in ≥ 1 segment (“narrow group”, *n* = 16). Patients with LAPW narrowing had a lower proportion of men, smaller echocardiographic LA size, and smaller 3D‐derived LAPW parameters (Table S1). ROC analysis showed no significant difference among the four LA parameters in discriminating LAPW narrowing (*p* = 0.779; Figure S1B and Table S2).

### 3.2 Left Atrial Posterior Wall Parameters After PVI in the Retrospective Phase

Representative LAPW voltage maps after PVI are shown in Figure 1. The distribution of LAPW narrowing (< 10 mm) across the three LAPW segments is shown in Figure 2A. LAPW narrowing was observed in the roof (5/52 [10%]), carina (14/52 [27%]), and bottom (4/52 [8%]) segments. Quantitative comparison demonstrated that the carina segment was significantly narrower than both the roof and the bottom. The mean band widths for the carina, roof, and bottom segments were 13.6 ± 7.1, 19.4 ± 7.9, and 23.6 ± 10.6 mm, respectively (carina vs. roof *p* < 0.001; carina vs. bottom *p* < 0.001; roof vs. bottom *p* = 0.005) (Figure 2B). ITD along the bilateral PVI lines strongly correlated with LAPW band width (TPI‐positive tags: *r* = 0.63, *p* < 0.001; all tags: *r* = 0.70, *p* < 0.001) (Figure 3A,B). These associations remained significant in additional mixed‐effects analyses (both *p* < 0.001). ROC analysis for predicting LAPW narrowing yielded an AUC of 0.869 for all‐tag ITD and 0.795 for TPI‐positive‐tag ITD; the AUC was numerically higher for all‐tag ITD, although the difference was not statistically significant (*p* = 0.099; Figure 3C). The optimal cutoffs were 16.5 mm and 24.9 mm, respectively.

**FIGURE 2:**
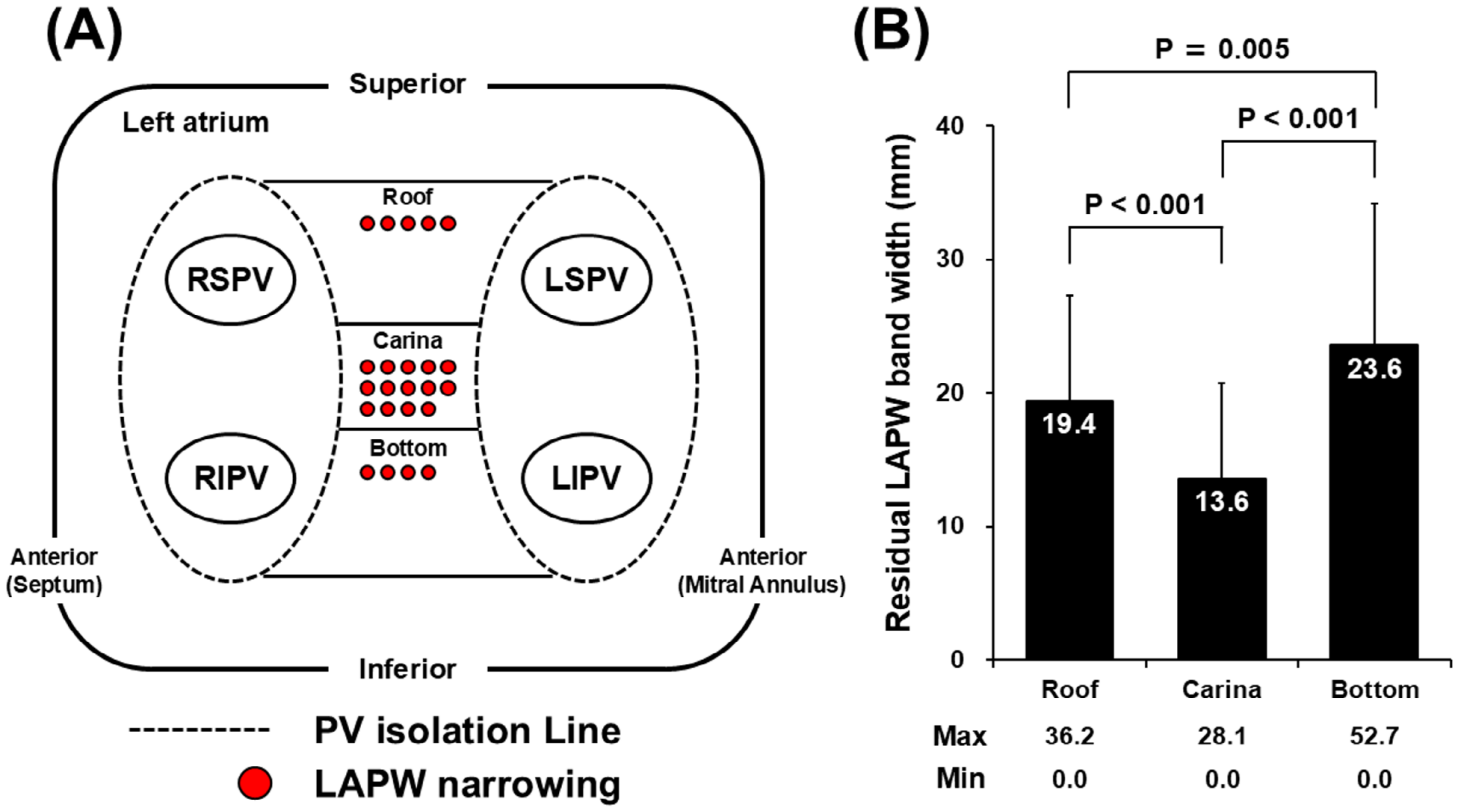
Distribution of LAPW narrowing and residual LAPW band width. (A) Schematic representation of LAPW segmentation (roof, carina, and bottom) and the distribution of LAPW narrowing (residual LAPW band width < 10 mm; red circles) after PVI. (B) Comparison of the residual LAPW band widths across segments. Data are presented as mean ± standard deviation. *p* values were derived from a linear mixed‐effects model accounting for within‐patient correlations, followed by Tukey‐adjusted pairwise comparisons. The maximum and minimum widths in each segment are shown below the corresponding bars. LAPW; left atrial posterior wall, PVI; pulmonary vein isolation.

**FIGURE 3:**
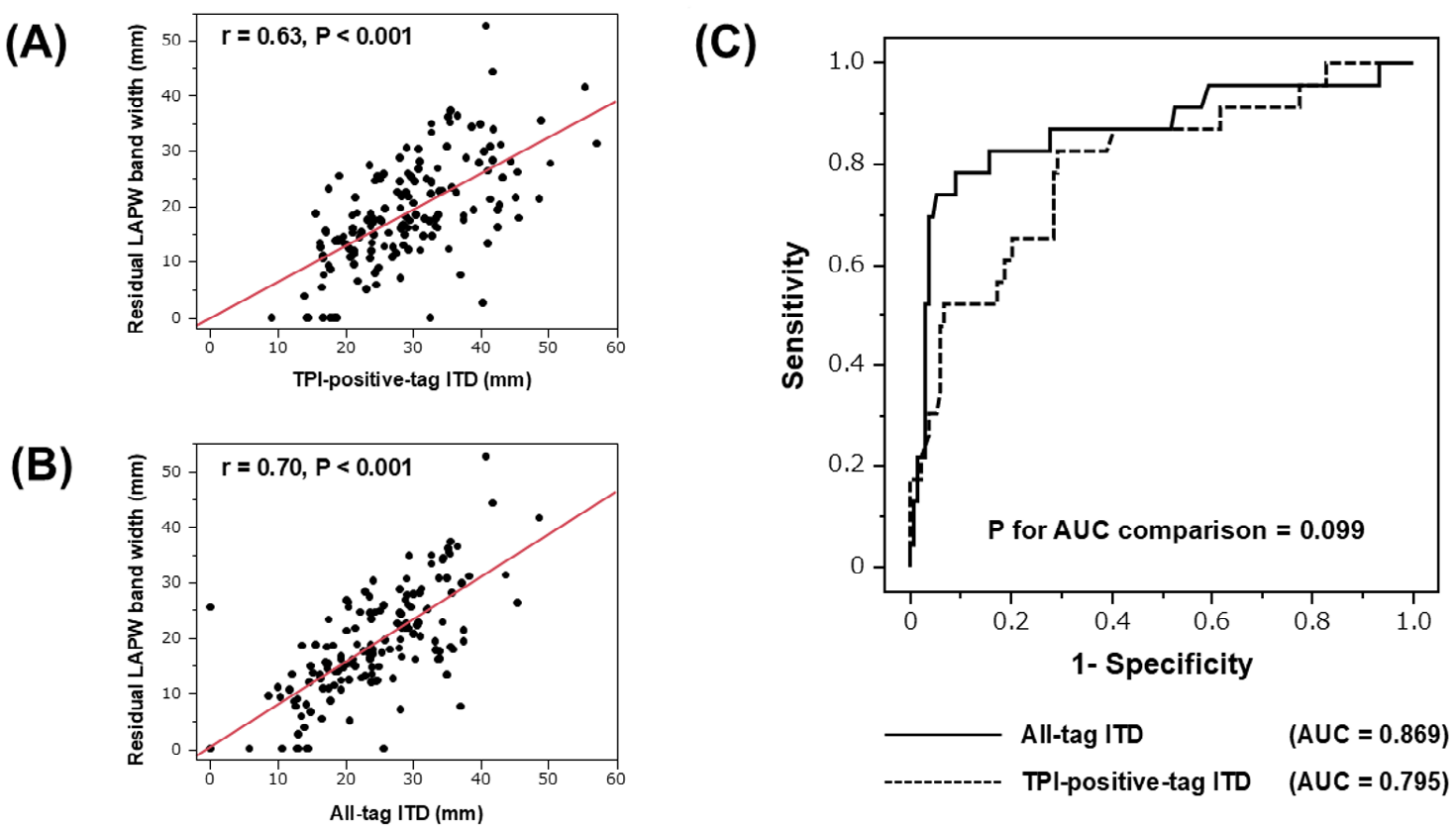
Association between inter‐tag distance and residual LAPW Band width. (A) Correlation between the TPI‐positive‐tag ITD and residual LAPW band width. (B) Correlation between all‐tag ITD and residual LAPW band width. (C) Receiver operating characteristic curves of all‐tag ITD and TPI‐positive‐tag ITD for discriminating LAPW narrowing (< 10 mm). LAPW; left atrial posterior wall, AUC, area under the curve, ITD, inter‐tag distance, TPI; tissue proximity indication.

### 3.3 Impact of ITD Guided Workflow on LAPW Preservation

Based on the retrospective analysis, all‐tag ITD showed a numerically stronger correlation with LAPW band width and a numerically higher AUC for predicting LAPW narrowing than TPI‐positive‐tag ITD. Therefore, the 16.5‐mm all‐tag ITD cutoff was adopted as the practical target in the prospective workflow (Figure 4). In the prospective phase, residual LAPW band width was assessed at the roof, carina, and bottom segments. Compared with the retrospective phase, the residual LAPW band was wider at the roof and carina, whereas no significant difference was observed at the bottom (roof: 19.4 ± 7.9 vs. 25.1 ± 7.1 mm, *p* = 0.002; carina: 13.6 ± 7.1 vs. 20.8 ± 8.0 mm, *p* < 0.001; bottom: 23.6 ± 10.6 vs. 26.0 ± 8.2 mm, *p* = 0.297; Figure 5A). Patients with at least one narrow LAPW band were numerically less frequent in the prospective than in the retrospective phase (3/27 [11%] vs. 16/52 [31%]; *p* = 0.053).

**FIGURE 4:**
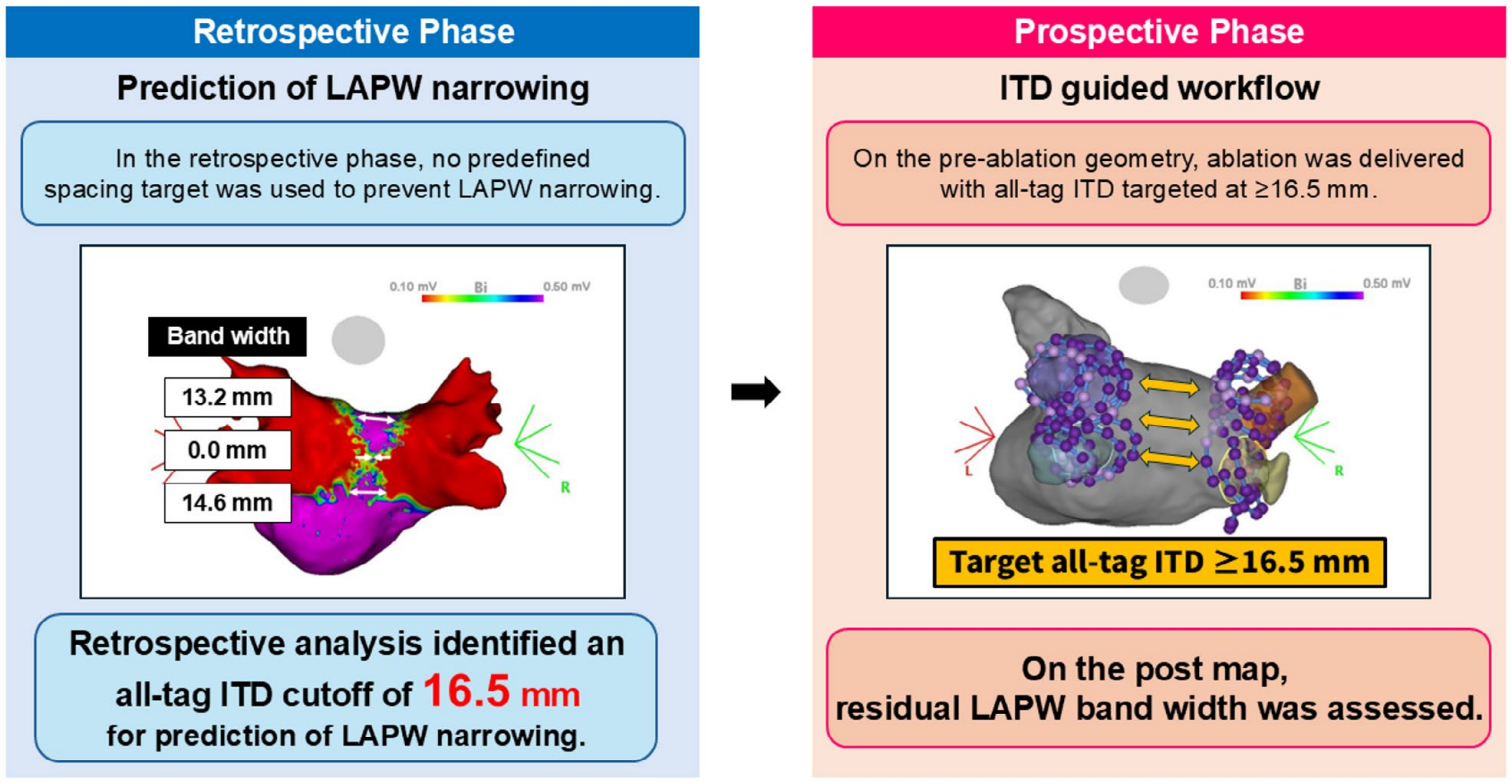
All‐tag ITD guided workflow for LAPW preservation. Retrospective analysis identified an all‐tag ITD cutoff of 16.5 mm for predicting LAPW narrowing. In the prospective phase, ablation was performed targeting an all‐tag ITD ≥ 16.5 mm on the pre‐ablation geometry, followed by assessment of residual LAPW band width on post‐ablation mapping. ITD, inter‐tag distance; LAPW, left atrial posterior wall.

**FIGURE 5:**
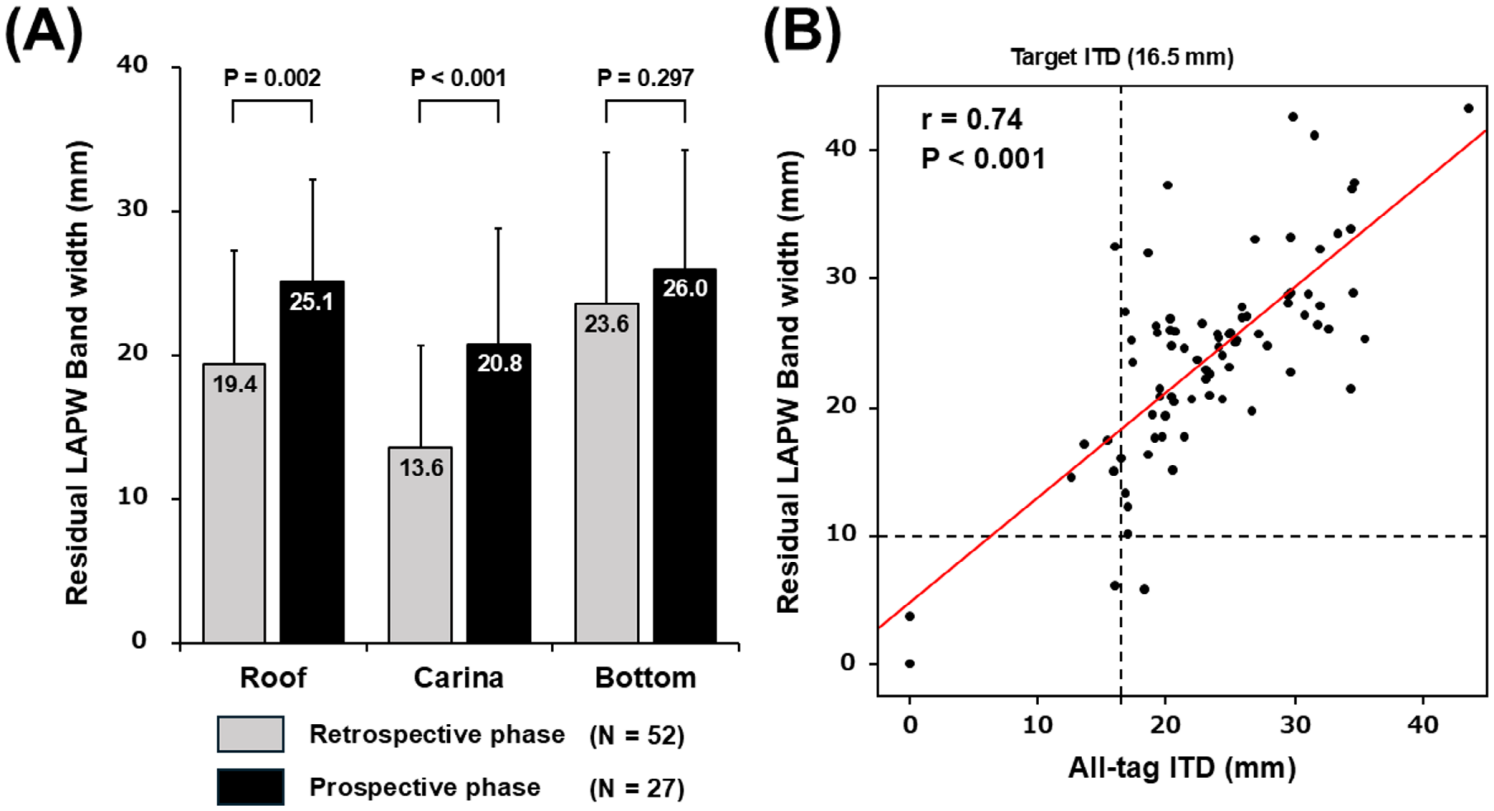
Impact of the ITD‐guided workflow on LAPW preservation. (A) Residual LAPW band widths at the roof, carina, and bottom in the retrospective and prospective phases. Data are presented as mean ± standard deviation. (B) Association between achieved all‐tag ITD and residual LAPW band width in the prospective phase. Dashed lines indicate the 16.5‐mm ITD target and 10‐mm narrowing threshold. LAPW, left atrial posterior wall; ITD, inter‐tag distance.

Among the 81 roof, carina, and bottom segments in the prospective phase, the mean achieved all‐tag ITD was 23.5 ± 7.2 mm, and the predefined target of ≥ 16.5 mm was achieved in 73 of 81 segments (90%). Achieved all‐tag ITD was strongly correlated with residual LAPW band width (*r* = 0.74, *p* < 0.001, Figure 5B). This association remained significant in an additional mixed‐effects analysis (*p* < 0.001). Four segments with LAPW narrowing were observed in 3 patients, and the predefined all‐tag ITD target was achieved in only 1 of 4 narrow segments (25%), compared with 72 of 77 non‐narrow segments (94%).

### 3.4 Follow Up in the Retrospective and Prospective Cohorts

During a median follow‐up of 248 days (IQR 185–365) in the retrospective phase, one patient was lost to follow‐up. Recurrent atrial tachyarrhythmias occurred in 6 of 51 patients (12%). Recurrence patterns included AF alone in 2 patients, AT alone in 1, and both AF and AT in 3. Atrial tachyarrhythmia recurrence did not differ between the LAPW narrow group (2/16, 13%) and the LAPW wide group (4/35, 11%) (*p* = 1.00). Of the two narrow‐group recurrences (including one patient who underwent LAPW isolation), one was AT and one was both AF and AT. The estimated 1‐year event‐free survival after the index procedure was 85.4% (95% confidence interval (CI), 69.3%–93.9%) (Figure 6).

**FIGURE 6:**
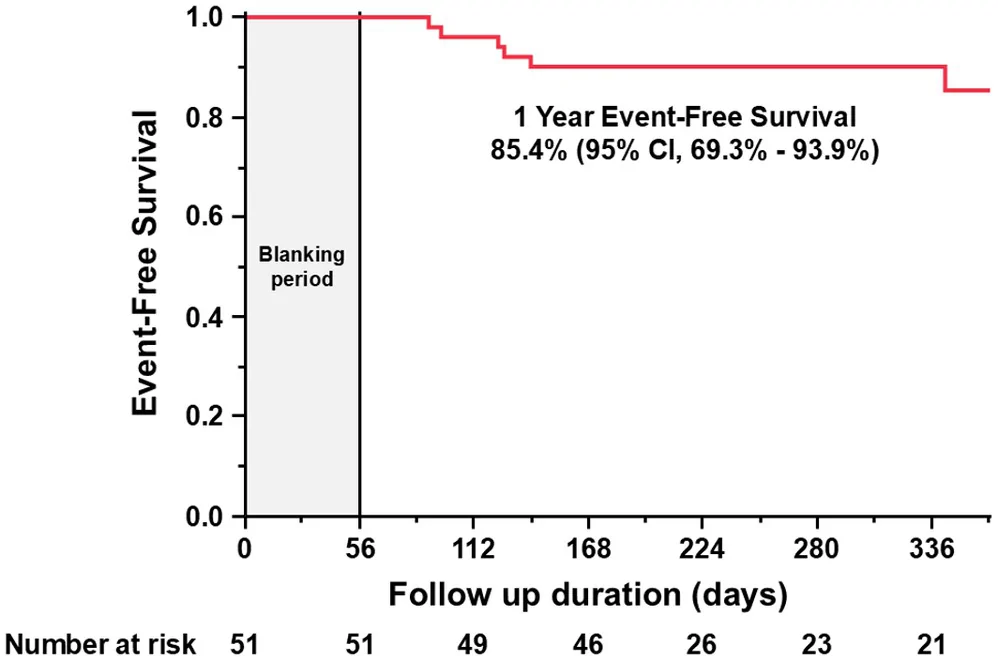
Kaplan–Meier curve of event‐free survival after the index procedure in the retrospective cohort.

In the prospective phase, the median follow‐up duration was 182 days (IQR, 103–184), and recurrent atrial tachyarrhythmias occurred in 5 of 27 patients (19%). All recurrent arrhythmias were AF. The estimated 6‐month event‐free survival was 81.5% (95% CI, 62.5%–92.1%; Figure S2).

### 3.5 Repeat‐Procedure Findings in the Retrospective Phase

Four patients (AF only, *n* = 1; AF + AT, *n* = 3) underwent repeat ablation, and conduction gaps along the PVI line were identified in all cases. Among the 3 patients with recurrent AT, one had an AT related to a conduction gap after previous superior vena cava isolation. In the remaining 2 patients, AF was the predominant arrhythmia during the repeat procedure, and the mechanism of AT could not be determined. No AT involving the LAPW was induced during the repeat procedures.

A representative repeat‐procedure case is shown in Figure 7A. The residual LAPW band width was wider on pre‐ablation mapping at the repeat procedure than on post‐ablation mapping at the index procedure (index vs. repeat: 23.9 ± 7.9 vs. 27.2 ± 5.2 mm; *p* = 0.038; *n* = 12 lines, Figure 7B). Segmentally, band width increased significantly at the roof (22.5 ± 5.3 vs. 28.1 ± 2.6 mm; *p* = 0.040; *n* = 4), but not at the carina (20.2 ± 7.2 vs. 23.0 ± 4.8 mm; *p* = 0.331; *n* = 4) and bottom (29.2 ± 9.5 vs. 30.6 ± 5.6 mm; *p* = 0.690; *n* = 4). Changes in residual LAPW band width were similar between lines with at least one TPI‐negative boundary tag and those with bilateral TPI‐positive boundary tags (median (IQR), 1.0 (−2.4 to 8.8) vs. 4.2 (0.6 to 6.9) mm; *p* = 1.00).

**FIGURE 7:**
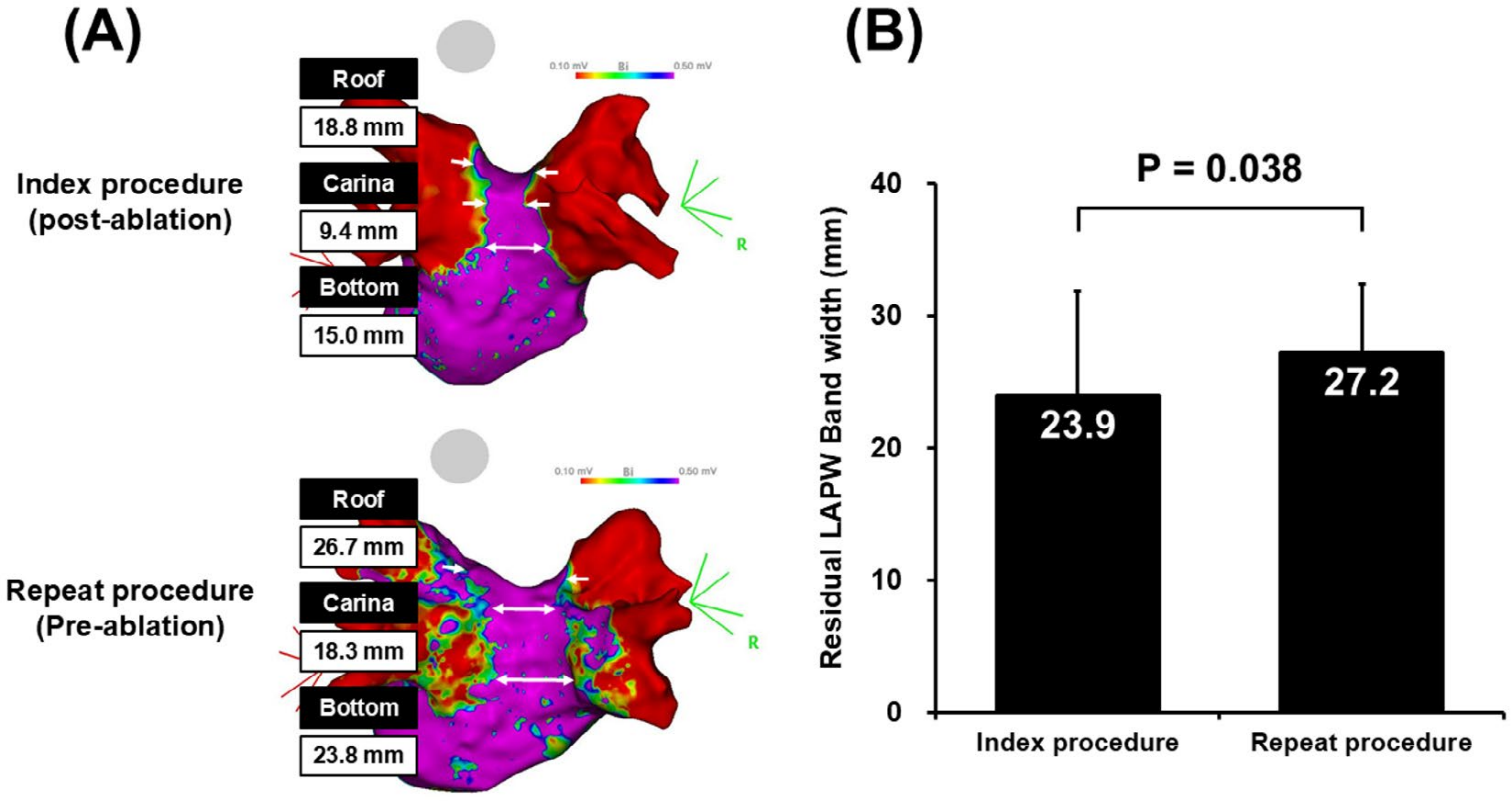
Changes in residual LAPW band width at repeat procedures. (A) Representative voltage maps after ablation at the index procedure and before ablation at the repeat procedure, showing widening of the residual LAPW band at the roof, carina, and bottom. (B) Comparison of residual LAPW band width between the index and repeat procedures in 12 corresponding lines from 4 patients. Data are presented as mean ± standard deviation, and the *p* value was derived from a paired Student's *t* test. LAPW, left atrial posterior wall; PV, pulmonary vein; TPI, tissue proximity indication.

### 3.6 Exploratory Mechanistic Analyses of Acute Lesion Characteristics

In an exploratory sub‐analysis of 5 patients, bipolar voltage was reduced at both TPI‐negative and TPI‐positive sites (TPI negative: pre 1.95 **±** 1.17 mV vs. post 0.54 **±** 0.55 mV; TPI positive: pre 1.44 **±** 0.96 mV vs. post 0.27 ± 0.26 mV; both, *p* < 0.001) (Figure S3A). In addition, analysis of covariance adjusting for pre‐voltage confirmed that post‐ablation voltage remained significantly higher at TPI‐negative than at TPI‐positive tags (adjusted LS‐means 0.488 mV [95% CI, 0.375–0.601] vs. 0.279 mV [95% CI, 0.227–0.331]; *p* = 0.001) (Figure S3B). Among TPI‐positive tags, adjusted post‐ablation voltage did not differ significantly across 1/3, 2/3, and 3/3 TPI‐positive deliveries (*p* = 0.097; Figure S4).

## 4 Discussion

### 4.1 Major Findings

This study has four major findings. First, all‐tag ITD and TPI‐positive‐tag ITD were strongly associated with residual LAPW band width. All‐tag ITD showed a numerically stronger correlation and higher discriminatory ability than TPI‐positive‐tag ITD. Second, incorporation of the retrospectively derived all‐tag ITD cutoff into the prospective workflow was associated with greater LAPW preservation. Third, repeat‐procedure voltage mapping suggested widening of the residual LAPW band, while the degree of widening did not differ according to the TPI status of the boundary tags at the index procedure. Fourth, exploratory mechanistic analysis showed acute voltage reduction at both TPI‐negative and TPI‐positive tags, although adjusted post‐ablation voltage remained higher at TPI‐negative tags.

### 4.2 TPI‐Related Lesion Distribution and LAPW Preservation

Posterior lesion extension after PFA with a pentaspline catheter has been reported, and narrow residual LAPW corridors may provide a substrate for macro‐reentrant AT [8]. In contrast, a prior VLCC study reported limited LAPW involvement, with no inadvertent LAPW isolation or need for additional ablation for residual slow‐conduction corridors [14]. However, in our retrospective phase, LAPW narrowing was observed in 16 of 52 patients and was associated with smaller LA size and 3D‐derived LAPW parameters, suggesting that patients with smaller LA anatomy may be more susceptible. Therefore, identifying procedural determinants of posterior lesion extension is important. Recently, proximal electrode heating was reported to be associated with excessive posterior lesion extension during PFA‐PVI using a lattice‐tip PFA catheter [18]. However, the determinants of unintended posterior lesion extension with a VLCC remain unclear. We therefore focused on the spatial distribution of TPI‐positive and all tags.

Both TPI‐positive tag and all‐tag ITD were strongly associated with residual LAPW band width, with all‐tag ITD showing numerically stronger correlation and discrimination. Incorporation of all‐tag ITD cutoff (16.5 mm) into the prospective workflow resulted in target achievement in 90% of LAPW segments and was associated with greater LAPW preservation, particularly at the roof and carina. Moreover, achieved all‐tag ITD was strongly associated with residual LAPW band width in the prospective cohort. Notably, the predefined target was achieved in only 1 of 4 (25%) segments with LAPW narrowing, compared with 72 of 77 (94%) non‐narrow segments. These findings suggest that an all‐tag ITD of 16.5 mm may serve as a practical target for avoiding excessive posterior lesion extension. Our exploratory mechanistic analysis further showed acute voltage reduction even at TPI‐negative tags, although adjusted post‐ablation voltage remained higher than at TPI‐positive tags. Thus, TPI‐negative ablations may still contribute to acute lesion modification, potentially explaining the numerically stronger association between all‐tag ITD and acute LAPW lesion extension. However, because the mechanistic analysis included only a small number of patients, these findings should be interpreted cautiously.

### 4.3 Chronic Regression of LAPW Lesions

Acute low voltage‐defined PFA lesions may not necessarily represent their persistent extent. Early regression of low‐voltage areas has been reported with both lattice‐tip and pentaspline PFA catheters [19, 20]. However, the chronic regression of LAPW lesions after PFA with a VLCC remains unclear. In our repeat‐procedure analysis, the residual healthy LAPW band was wider at the repeat procedure than at the index procedure, suggesting potential regression of the acute lesion extent over time (Figure 7B). Interestingly, the degree of widening did not differ according to the TPI status of the boundary tags at the index procedure. These findings suggest that acute LAPW narrowing may not necessarily persist chronically and that TPI status alone may not predict chronic lesion regression. Additionally, no LAPW‐related AT was identified or induced during the repeat procedures. These observations suggest that additional LAPW ablation solely because of acute LAPW narrowing should be considered cautiously, with clinical and electrophysiological follow‐up as an alternative approach. However, these findings were derived from an exploratory analysis of only 4 patients undergoing repeat procedures and should therefore be interpreted cautiously. Further studies in larger cohorts with systematic repeat mapping are needed to clarify the chronic regression of LAPW lesions.

### 4.4 Clinical Implications

These findings suggest that an all‐tag ITD of 16.5 mm may provide a practical intraprocedural target when attempting to limit unintended LAPW narrowing during VLCC‐based PFA, particularly in patients with smaller atrial anatomy. Acute LAPW narrowing alone may not necessarily indicate the need for additional LAPW ablation, given the possibility of chronic lesion regression, and careful clinical and electrophysiological follow‐up may be a reasonable alternative.

### 4.5 Study Limitations

This study has several limitations. First, the patient population was relatively small, which reduces statistical power and precision. Second, our observations come from procedures using a VLCC on the CARTO 3 platform within a specific workflow; generalizability to other PFA systems is uncertain. Third, the repeat‐procedure and mechanistic analyses were based on only 4 and 5 patients, respectively, and should therefore be considered exploratory. Because of the limited sample sizes, these site‐ and tag‐level analyses were performed without adjustment for within‐patient clustering, and their findings should be interpreted cautiously. Fourth, several procedural factors differed between the retrospective and prospective phases, including irrigation rate, mapping workflow, CARTO 3 version, and operator experience; therefore, the greater LAPW preservation observed prospectively may have been influenced by these factors in addition to the all‐tag ITD workflow. Finally, follow‐up in the prospective phase was relatively short, limiting the assessment of long‐term clinical outcomes and chronic lesion durability.

## 5 Conclusion

In this study evaluating PFA with a VLCC, incorporation of a retrospectively derived all‐tag ITD cutoff of 16.5 mm into the prospective workflow was associated with greater LAPW preservation. These findings suggest that all‐tag ITD may serve as a practical intraprocedural target for limiting unintended LAPW narrowing during VLCC‐based PFA.

## Disclosure

During manuscript preparation, the authors used ChatGPT (OpenAI; used during 2025–2026; model versions not recorded) for English‐language editing and improvements to clarity and organization throughout the manuscript. The authors reviewed all AI‐assisted text against the original manuscript to ensure accuracy and preservation of the intended meaning. No identifiable patient information was entered into the tool. The authors take full responsibility for the final content.

## Ethics Statement

This study was conducted according to the Declaration of Helsinki and approved by the Institutional Review Board of Nihon University Itabashi Hospital (RK‐240312‐1).

## Consent

Informed consent was obtained using an opt‐out approach in accordance with institutional and ethical requirements.

## Conflicts of Interest

Dr. Okumura has received speaker honoraria from AstraZeneca K.K. and Johnson & Johnson K.K.; research funding from MEDTRONIC JAPAN CO. LTD., MicroPort CRM Japan, and Bayer Healthcare; and endowed chair support from Abbott Japan LLC, Boston Scientific Japan K.K., MEDTRONIC JAPAN CO. LTD., Japan Lifeline Co. Ltd., and BIOTRONIK. Dr. Nagashima has received speaker honoraria from Johnson & Johnson/Biosense Webster, Medtronic Japan, and Boston Scientific Japan. Other authors have no conflicts of interest for this article.

## Supporting information


**Figure S1:** 3D mapping‐based assessment of LAPW anatomy and ROC curves for LAPW narrowing. (A) Representative pre‐ablation 3D mapping geometry illustrating measurement of LAPW surface area and the roof and bottom widths. Mean anatomical LAPW width was defined as the average of the roof and bottom widths. (B) Receiver operating characteristic curves for mean anatomical LAPW width, LAPW surface area, left atrial volume, and left atrial diameter for predicting LAPW narrowing (< 10 mm). Comparison of the four AUCs showed no significant difference (overall *p* = 0.779). AUC, area under the curve; LAPW, left atrial posterior wall.
**Figure S2:** Kaplan–Meier curve of event‐free survival in the prospective cohort.
**Figure S3:** Changes in bipolar voltage from pre‐ to post‐ablation and adjusted post‐ablation voltage by TPI status. (A) Upper panels: representative left atrial bipolar voltage maps before and after a single ablation set, with TPI‐negative and TPI‐positive tags indicated; arrowheads denote TPI‐negative tags. Lower panels: bar plots show mean ± standard deviation bipolar voltage at the corresponding tag locations on pre‐map and post‐map for TPI‐negative tags (*n* = 31) and TPI‐positive tags (*n* = 141). Both groups demonstrated significant reductions (paired Student's *t*‐tests, *p* < 0.001). (B) Post‐map bipolar voltage by TPI status from analysis of covariance with pre‐ablation voltage as a covariate. Points show adjusted LS‐means and error bars show 95% confidence intervals. LS; least squares, TPI; tissue proximity indication.
**Figure S4:** Adjusted post‐ablation bipolar voltage according to the number of TPI‐positive energy deliveries. Among TPI‐positive tags, post‐ablation bipolar voltage was compared among sites with TPI positivity during 1, 2, or all 3 energy deliveries within a single ablation set. Points indicate adjusted least‐squares means derived from analysis of covariance with pre‐ablation voltage as a covariate, and error bars indicate 95% confidence intervals. Adjusted post‐ablation voltage did not differ significantly across 1/3, 2/3, and 3/3 TPI‐positive deliveries (adjusted LS‐means 0.237 mV [95% CI, 0.108–0.367], 0.240 mV [95% CI, 0.188–0.292], and 0.339 mV [95% CI, 0.263–0.414], respectively; *p* = 0.097). LS, least squares; TPI, tissue proximity indication.
**Table S1:** Baseline characteristics in retrospective phase.
**Table S2:** Diagnostic performance of echocardiographic and 3D mapping‐derived anatomical parameters for predicting LAPW narrowing.

## Data Availability

The data supporting the findings of this study are not available.
