# Supplementary material for: Posterior Pulmonary Vein Isolation Lesion Spacing and Left Atrial Posterior Wall Narrowing During Pulsed Field Ablation
Source: J Arrhythm. 2026 Oct 28;42(6):e70509. doi: 10.1002/joa3.70509 (PMC13715061; doi:10.1002/joa3.70509)
Supplement: Supplementary file 1 [file JOA3-42-e70509-s001.docx]

**SUPPLEMENTARY MATERIALS**

**Posterior Pulmonary Vein Isolation Lesion Spacing and Left Atrial Posterior Wall Narrowing During Pulsed Field Ablation**

Yuji Saito^1^, MD, Koichi Nagashima^1^, MD, Ryuta Watanabe^1^, MD, Naoto Otsuka^1^, MD, Shu Hirata^1^, MD, Moyuru Hirata^1^, MD, Masanaru Sawada^1^, MD, Hikaru Masuda^1^, MD, Akinori Matsushima^1^, MD, Masato Sakaki^1^, MD, and Yasuo Okumura^1^, MD

**Author affiliations**

1. Division of Cardiology, Department of Medicine, Nihon University School of Medicine, Tokyo, Japan.

Corresponding author: Yasuo Okumura, MD

Division of Cardiology, Department of Medicine, Nihon University School of Medicine

Ohyaguchi-kamicho, Itabashi-ku, Tokyo 173-8610, Japan

E-mail: [okumura.yasuo@nihon-u.ac.jp](mailto:okumura.yasuo@nihon-u.ac.jp)

**Short title:** All-tag ITD Guided Workflow during VLCC-PFA

**Supplementary Figure S1**

**3D mapping-based assessment of LAPW anatomy and ROC curves for LAPW narrowing.**

(A) Representative pre-ablation 3D mapping geometry illustrating measurement of LAPW surface area and the roof and bottom widths. Mean anatomical LAPW width was defined as the average of the roof and bottom widths. (B) Receiver operating characteristic curves for mean anatomical LAPW width, LAPW surface area, left atrial volume, and left atrial diameter for predicting LAPW narrowing (<10 mm). Comparison of the four AUCs showed no significant difference (overall P = 0.779). AUC, area under the curve; LAPW, left atrial posterior wall.

**Supplementary Figure S2.**

**Kaplan–Meier curve of event-free survival in the prospective cohort.**

**Supplementary Figure S3.**

**Changes in bipolar voltage from pre- to post-ablation and adjusted post-ablation voltage by TPI status.**

(A) Upper panels: representative left atrial bipolar voltage maps before and after a single ablation set, with TPI-negative and TPI-positive tags indicated; arrowheads denote TPI-negative tags. Lower panels: bar plots show mean ± standard deviation bipolar voltage at the corresponding tag locations on pre-map and post-map for TPI-negative tags (n = 31) and TPI-positive tags (n = 141). Both groups demonstrated significant reductions (paired Student’s t-tests, P < 0.001). (B) Post-map bipolar voltage by TPI status from analysis of covariance with pre-ablation voltage as a covariate. Points show adjusted LS-means and error bars show 95% confidence intervals. LS; least squares, TPI; tissue proximity indication.

**Supplementary Figure S4.**

**Adjusted post-ablation bipolar voltage according to the number of TPI-positive energy deliveries.**
Among TPI-positive tags, post-ablation bipolar voltage was compared among sites with TPI positivity during 1, 2, or all 3 energy deliveries within a single ablation set. Points indicate adjusted least-squares means derived from analysis of covariance with pre-ablation voltage as a covariate, and error bars indicate 95% confidence intervals. Adjusted post-ablation voltage did not differ significantly across 1/3, 2/3, and 3/3 TPI-positive deliveries (adjusted LS-means 0.237 mV [95% CI, 0.108-0.367], 0.240 mV [95% CI, 0.188-0.292], and 0.339 mV [95% CI, 0.263-0.414], respectively; P = 0.097). LS, least squares; TPI, tissue proximity indication.

**Supplementary Table S1. Baseline characteristics in retrospective phase**

|  | Total  (n=52) | Wide group  (n=36) | Narrow group  (n=16) | P value |
| --- | --- | --- | --- | --- |
| Clinical characteristics |  |  |  |  |
| Age (years) | 64 ± 11 | 64 ± 12 | 65 ± 8 | 0.718 |
| Male sex | 38 (73%) | 30 (83%) | 8 (50%) | 0.019 |
| Body mass index (kg/m^2^) | 24 ± 5 | 24 ± 5 | 22 ± 3 | 0.165 |
| Paroxysmal AF | 45 (87%) | 30 (83%) | 15 (94%) | 0.415 |
| AF duration (months) | 5 (3.3, 14) | 5 (3, 14.8) | 5.5 (4, 9.5) | 0.905 |
| Hypertension | 25 (48%) | 16 (44%) | 9 (56%) | 0.432 |
| Dyslipidemia | 15 (29%) | 9 (25%) | 6 (38%) | 0.508 |
| Diabetes mellitus | 10 (19%) | 6 (17%) | 4 (25%) | 0.475 |
| Heart failure | 11 (21%) | 10 (28%) | 1 (6%) | 0.140 |
| History of stroke | 4 (8%) | 3 (8%) | 1 (6%) | 1.00 |
| Vascular disease | 1 (2%) | 1 (3%) | 0 (0%) | 1.00 |
| CHADS_2_ score | 1 (0, 2) | 1 (0.3, 2) | 1 (0, 1.8) | 0.477 |
| CHA_2_DS_2_-VASc score | 2 (1, 3) | 2 (1, 3) | 2 (0.3, 3) | 0.855 |
| Antiarrhythmic drug use | 27 (52%) | 17 (47%) | 10 (63%) | 0.309 |
| Class I | 16 (31%) | 10 (28%) | 6 (38%) | 0.527 |
| Class III | 2 (4%) | 2 (6%) | 0 (0%) | 1.00 |
| Class IV | 11 (21%) | 5 (14%) | 6 (38%) | 0.073 |
| Echocardiographic measurements |  |  |  |  |
| Left ventricular ejection fraction (%) | 67 ± 9 | 68 ± 8 | 66 ± 10 | 0.555 |
| Left atrial diameter (mm) | 38 ± 6 | 39 ± 6 | 35 ± 7 | 0.027 |
| Left atrial volume (mL) | 43 ± 19 | 46 ± 19 | 35 ± 16 | 0.046 |
| 3D Mapping-Based LAPW Assessment |  |  |  |  |
| LAPW surface area (cm^2^) | 18.1 ± 4.6 | 19.4 ± 4.2 | 15.2 ± 4.2 | 0.002 |
| Mean anatomical LAPW width (mm) | 40.2 ± 7.1 | 42.2 ± 6.1 | 35.5 ± 7.0 | 0.001 |
| First-pass PV isolation success | 48 (92%) | 35 (97%) | 13 (81%) | 0.081 |
| Number of ablations before post-mapping | 23 ± 5 | 24 ± 5 | 23 ± 4 | 0.520 |
| Number of applications before post-mapping | 66 ± 13 | 67 ± 14 | 63 ± 11 | 0.298 |
| Number of total ablations | 24 ± 6 | 24 ± 5 | 25 ± 6 | 0.372 |
| Number of total applications | 69 ± 16 | 68 ± 15 | 71 ± 17 | 0.599 |
| Total fluoroscopic time (min) | 0.0 (0.0, 0.2) | 0.0 (0.0, 1.6) | 0.0 (0.0, 0.0) | 0.154 |
| Total fluoroscopic dose (mGy) | 0.0 (0.0, 0.7) | 0.0 (0.0, 5.5) | 0.0 (0.0, 0.1) | 0.595 |
| Primary PV isolation time (min) | 31 ± 10 | 30 ± 10 | 31 ± 10 | 0.871 |
| Total procedure time (min) | 107 ± 29 | 106 ± 29 | 109 ± 28 | 0.734 |

Values are shown as the mean ± standard deviation, median (25th, 75th interquartile range) or n (%). AF, atrial fibrillation; LAPW, left atrial posterior wall; PV, pulmonary vein.

**Supplementary Table S2. Diagnostic performance of echocardiographic and
3D mapping-derived anatomical parameters for predicting LAPW narrowing.**

AUC, area under the curve; CI, confidence interval; LAPW, left atrial posterior wall.

| Parameter | AUC (95%CI) | Optimal cutoff | Sensitivity (%) | Specificity (%) |
| --- | --- | --- | --- | --- |
| Echocardiographic parameters |  |  |  |  |
| Left atrial diameter (mm) | 0.699 (0.507 - 0.839) | 34.4 | 56.3 | 80.6 |
| Left atrial volume (mL) | 0.717 (0.530 - 0.851) | 30.8 | 56.3 | 88.9 |
| 3D mapping parameters |  |  |  |  |
| LAPW surface area (cm^2^) | 0.772 (0.583 - 0.891) | 15.6 | 75.0 | 80.6 |
| Mean anatomical LAPW width (mm) | 0.787 (0.602 - 0.901) | 36.1 | 68.8 | 86.1 |
